# Ocular Involvement of Granulomatosis with Polyangiitis

**DOI:** 10.3390/jcm12134448

**Published:** 2023-07-02

**Authors:** Anna Byszewska, Izabela Skrzypiec, Aleksandra Rymarz, Stanisław Niemczyk, Marek Rękas

**Affiliations:** 1 Ophthalmology Department, Military Institute of Medicine–National Research Institute, Szaserów 128, 04-141 Warsaw, Poland; 2 Nephrology Department, Military Institute of Medicine–National Research Institute, Szaserów 128, 04-141 Warsaw, Poland; arymarz@wim.mil.pl (A.R.);

**Keywords:** GPA, Wegener’s disease, GPA ocular symptoms, ANCA, vasculitis

## Abstract

Granulomatosis with polyangiitis (GPA), formerly referred to as Wegener’s disease, is a form of ANCA-associated vasculitis. It manifests mainly in the kidneys and the upper respiratory tract, but ocular involvement is not uncommon. In this article, four cases with ocular manifestations are presented with comprehensive photographic documentation. We describe the way to proper diagnosis, which may be long, the possible treatment, and the final outcomes. Our patients had the following ocular manifestations of GPA: retinal vasculitis, anterior necrotizing scleritis, medial orbital wall and orbital floor erosion with middle face deformation, compressive optic neuropathy due to retrobulbar inflammatory mass, and the abscess of the eyelids, inflammatory intraorbital mass causing exophthalmos and diplopia. This manuscript includes the description of severe forms of GPA, the initial signs and symptoms, relapses, and difficulties in achieving remission. The extraocular involvement is described with diagnostic modalities and laboratory findings. One of the reported cases was diagnosed by an ophthalmologist on the basis of ocular symptoms in the early stages of the disease. Our outcomes are compared with those discussed in the literature.

## 1. Introduction

Granulomatosis with polyangiitis (GPA, formerly referred to as Wegener’s granulomatosis) is a rare systemic disease belonging to small vessel vasculitides. According to the Chapel Hill classification, GPA, together with microscopic polyangiitis (MPA) and eosinophilic GPA (EGPA), belong to the group of ANCA (antineutrophil cytoplasmic antibodies)-associated vasculitis (AAV) [1]. This group of diseases affects small and medium vessels of numerous regions in the body. An inflammatory state destroys the vessel wall and impairs blood flow, leading to ischemia and even to the necrosis of the organ supplied by the vessel. ANCA plays a crucial role in the development of this disease, but ANCA-negative cases of the disease may also occur [2]. The majority of patients with GPA have antibodies against proteinase 3 (PR-3 ANCA) [3].

Many regions of the body may be affected by this disease, but the typical course of GPA is associated with the following disturbances: upper respiratory tract inflammation with sinusitis and nasal lesions, pulmonary infiltrations, and glomerulonephritis [4,5]. The prevalence of ocular manifestations of the disease is 50%, with 8–16% of cases developing them as the first manifestation [6,7,8,9].

Numerous possible ocular symptoms of the disease may include episcleritis, scleritis, conjunctivitis, keratitis, uveitis, retinal vasculitis, retinal arterial or venous thrombosis, retinal exudates, retinal hemorrhages, blurred vision, blindness, proptosis and orbital granulomatous masses, and epiphora [10].

The proper diagnosis at the onset of symptoms is associated with better chances of recovery and limits the destruction of the affected organs. The diversity of the symptoms frequently makes the diagnosis very difficult, which leads to a delay in proper treatment. In almost one-third of patients, the delay in diagnosis exceeds six months [11]. Immunosuppressive therapy is necessary to stop the inflammatory process and avoid tissue damage and organ dysfunction.

The organ-limited form of the disease may be treated with methotrexate (MTX) or mycophenolate mofetil (MMF) with concomitant glucocorticosteroids (GCs). The life- or organ-threatening course of the disease requires more intensive immunosuppressive treatment, which is composed of two phases. The first one is induction therapy with higher doses of the drugs, and the second phase is maintenance therapy. The first one lasts about 6 months, and the second one should last no less than 2 years. In the induction therapy, cyclophosphamide (CYC) or rituximab (RTX) is used with concomitant GCs, whereas maintenance therapy comprises RTX, MTX, azathioprine (AZA), MMF, and GCs [12].

ANCA-associated vasculitides are rare diseases with the prevalence estimated at 20 per 1 mln per 1 year in Europe. Given the Polish population of ca 37 million people, there should be about 700 new cases each year.

In this article, we describe GPA clinical presentations with photographic documentation describing the long and difficult path to the proper diagnosis. We also discuss difficulties in maintaining remission, as the disease is characterized by multiorgan involvement, and its course is mostly severe.

The awareness of GPA among ophthalmologists is necessary for daily clinical practice to establish the right diagnosis and to refer the patient to the proper specialist, who may offer adequate immunosuppressive therapy.

This paper includes clinical pictures of four patients with GPA with ocular involvement, presented from an ophthalmologist’s point of view. One of the patients was diagnosed by an ophthalmologist and referred for immunosuppressive therapy by a cooperating nephrologist. Patients with a previously established diagnosis were consulted in the Ophthalmology Department.

The success of the long life expectancy of the presented patients largely depends on the involvement of a multidisciplinary team with the leading role of an experienced immunologist. Numerous cases with ophthalmic manifestations were described in the literature, where the final outcomes cannot be assessed because the patients passed away due to extraocular organ failure [13,14]. Those most severe clinical situations were associated with a long time passing between the onset of the symptoms and the assessment and the late establishment of the diagnosis.

## 2. Methods

In this paper, we present a clinical picture of patients with GPA referred to the Ophthalmology Department of the Military Institute of Medicine–National Research Institute in Warsaw. All patients signed the informed consent for treatment and consented to photographic documentation being taken and its use for publication. The photographs were taken during consultations or provided by the patients themselves. The initial signs and symptoms and the course of the disease, treatment modalities, and final outcome are described.

The World Medical Association Declaration of Helsinki and the principles developed by the European Union entitled Good Clinical Practice for Trials on Medical Products in the European Community were followed in this study.

## 3. Main Part

### 3.1. Case 1

Clinical presentation includes the involvement of corneal ulcers, anterior necrotizing scleritis, and retinal vasculitis.

**Figure 1:**
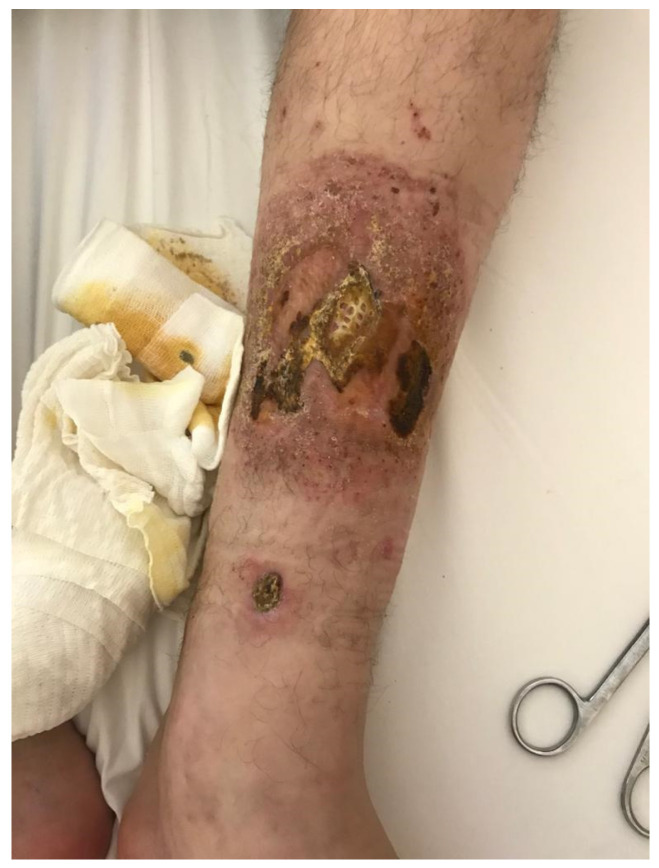
Left lower limb skin ulcer before the introduction of treatment.

A 40-year-old patient with mild mental retardation was admitted to the Trauma and Orthopedics Department in our facility due to severe skin ulceration, which caused difficulties walking due to severe pain in his left lower limb along the left shin (Figure 1). The condition did not improve despite surgical treatment and specialized bandages. Active infectious foci in the teeth, 13 in total, were suggested as the underlying cause for the ulceration. Following radiological confirmation, all the infected teeth were removed.

The left lower limb ulcer was surgically debrided, and the patient was discharged. He returned after a month, and the condition of his wound deteriorated. It was covered with necrotic tissue to a larger extent. New ulcers with granulomatosis appeared as follows: much smaller ones on the sole, in the mouth, on the hand, and on the fingers. They were also difficult to cure. The patient was referred to a hematologist with suspicion of immunodeficiency due to recurring necrotic foci in different locations. He underwent a bone marrow biopsy, the result of which was unremarkable. He was suspected of acquired immunodeficiency incompetence.

After 4 months, during another hospitalization, he had discomfort in his right eye, so he was referred to the Ophthalmology Department for assessment. His visual acuity was logMar 0.1 in the right eye (RE) and 0.0 in the left eye (LE). His intraocular pressure (IOP) was 10 and 11 mmHg, respectively.

No photophobia or pain was reported.

**Figure 2:**
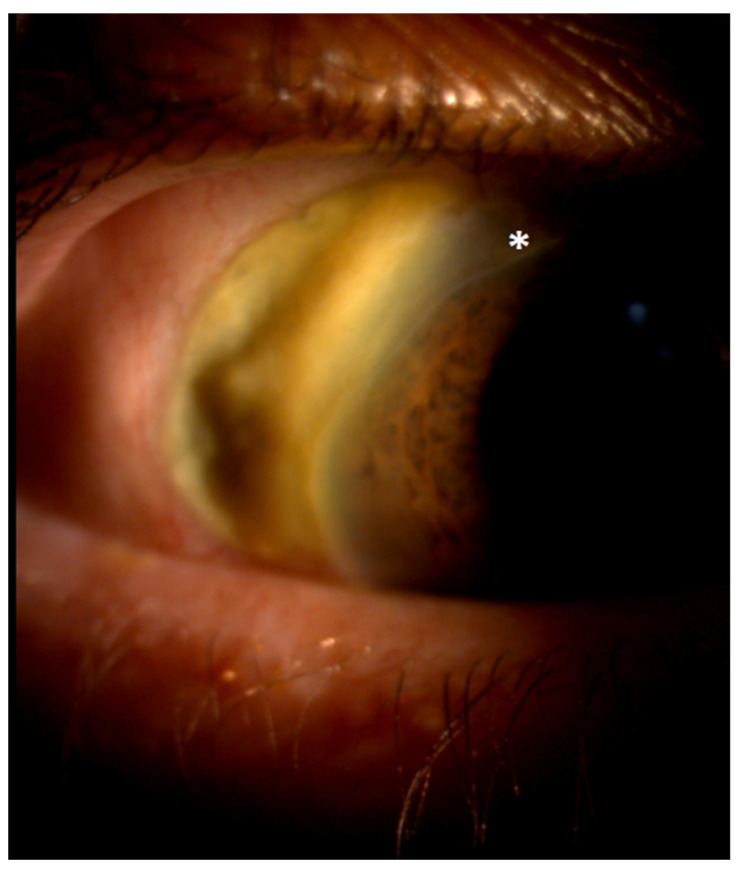
A photo of the patient’s right eye at the initial presentation. Conjunctival redness and chemosis. Adjacent to the area of scleritis, corneal peripheral keratitis can be distinguished (the asterisk).

**Figure 3:**
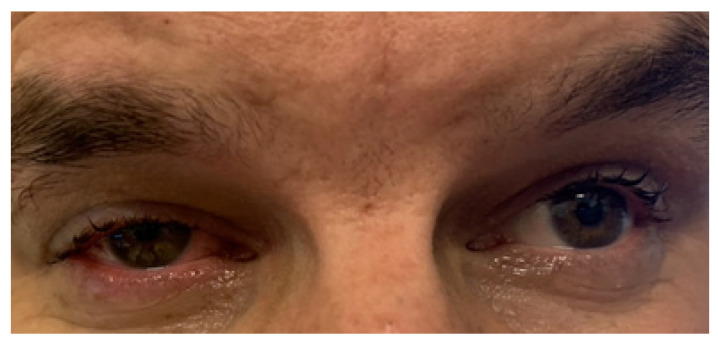
External photo at the initial presentation.

**Figure 4:**
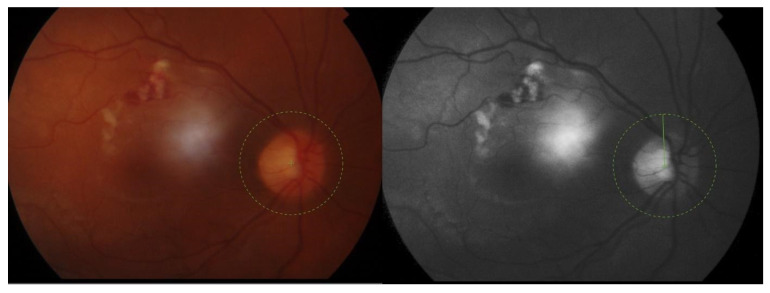
A photo of the fundus with vasculitis in the retinal vessels. Arteries and veins are involved. Cotton wool spots and hemorrhages are present. A color photo on the left and a red-free photo on the right.

Conjunctival redness was observed in the RE. A slit lamp examination revealed necrotizing anterior scleritis (Figure 2 and Figure 3) with the exposed choroid, covered with very thin or no sclera, chemosis, and the redness of adjacent conjunctiva from 8 to 12 o’clock. On the adjacent cornea, along 8-1 o’clock, a marginal ulcer was present (characteristic of GPA peripheral ulcerative keratitis) with no inflammation in the anterior chamber. In the posterior segment of the RE, the following signs of vasculitis were present along the upper temporal arcade: multiple cotton wool spots with flame hemorrhages, hemorrhages in the macular region, tortuous arteries, and dilated veins (Figure 4). 

**Figure 5:**
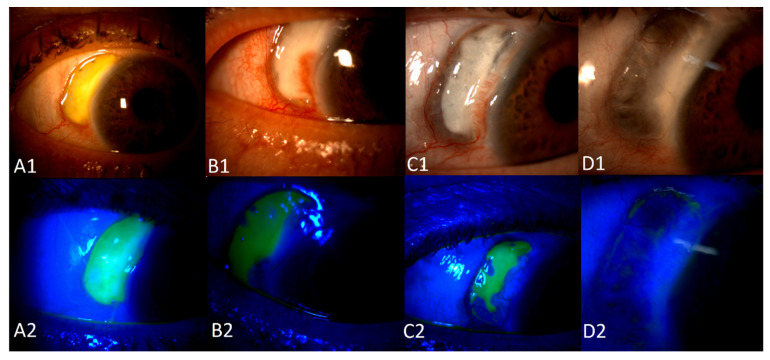
Anterior necrotizing scleritis. The upper image is slit lamp photo, and the lower image is a photo with fluorescein and a cobalt filter. (**A1**) Initial presentation, instead of the sclera, a whitish discharge overlies the uvea. (**A2**) Fluorescein staining in the whole area of scleritis. (**B1**) New vessels are seen around the margins of scleritis. (**B2**) No staining in the areas of new vessels. (**C1**) More new vessels around the area. (**C2**) A significantly smaller area of staining. The area without staining is covered with new tissue. (**D1**) After recovery, the white tissue with a net of small vessels covers the uvea. (**D2**) No staining.

Anterior necrotizing scleritis was diagnosed.(Figure 5).

Differential diagnoses included granulomatosis with polyangiitis, rheumatoid arthritis, arthritis, cytomegalovirus infection (CMV), and acquired immunodeficiency. In the meantime, blood cell disease was excluded by a hematologist with an unremarkable bone marrow biopsy.

The biopsy of granuloma in the soft palate was collected for histopathological assessment.

The laboratory work up was positive for cANCA and negative for pANCA. Other investigations showed COVID (-), a human immunodeficiency syndrome (HIV) (-), elevated IgG and IgE titers, decreased C4 complement, hypoalbuminemia, and normocytic anemia with elevated platelets (possible signs of chronic disease).

At the initial phase, local fluoroquinolone, non-steroidal anti-inflammatory agents (bromfenac eye drops), loteprednol eye drops (topical GCs), and artificial tears were prescribed as treatment for anterior necrotizing scleritis. The patient was referred to the Nephrology Department, where he received systemic GCs at a dose of 60 mg daily and was started on immunosuppressive therapy with 6 monthly doses of CYC (according to the CYCLOPS protocol).

Following the introduction of local GCs, the deterioration of peripheral ulcerative keratitis and scleritis was noted. The GCs were discontinued. The patient was assessed as being at a high risk of perforation and was initially qualified for the surgical covering of the wound with a scleral patch. The introduction of suitable, aggressive systemic treatment of GPA helped to limit severe ocular manifestations as well as lesions in other parts of the body, as described above. 

Eventually, the patient did not require a scleral patch (Figure 5). From an ophthalmologist’s clinical point of view, local GCs are contraindicated in GPA scleritis.

The remission of ophthalmic symptoms was due to adequate immunosuppressive therapy introduced by other physicians from a multidisciplinary team at our facility.

### 3.2. Case 2

The second case involves the orbit and the middle face, diplopia, erosion of the medial wall and orbital floor, bare medial and inferior extraocular muscles, loss of the conjunctiva, lagophthalmos, epiphora, and enophthalmos.

A 43-year-old female was referred to the Nephrology Department with the diagnosis of GPA. She had a history of left mastectomy due to breast cancer, followed by chemotherapy and radiotherapy. She still takes tamoxifen. Subsequently, she had a lung tumor resection with wound healing difficulties. The biopsy of the wound tissues led to the diagnosis of GPA.

**Figure 6:**
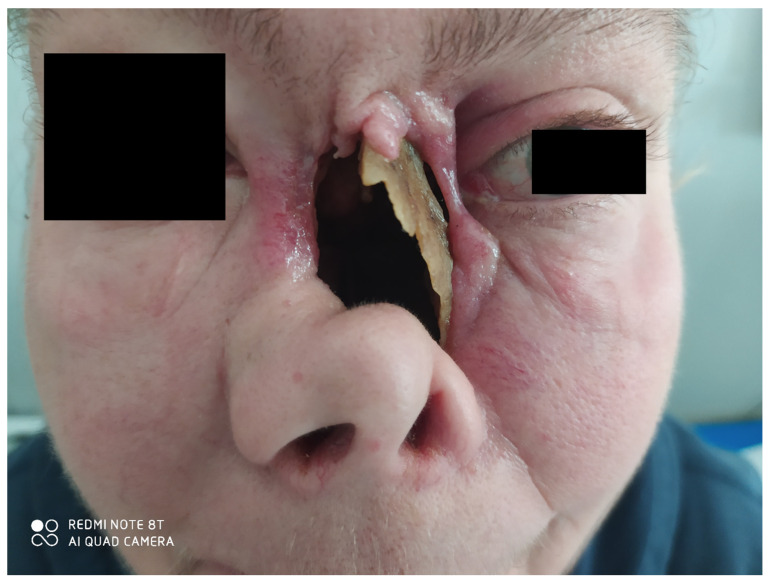
The collapsed nasal cavity with the involvement of the left orbit. Necrotic nasal structures. The position of the lower eyelid is lower than in the right eye. Lack of soft tissues in the median palpebral angle.

The activity of the disease encompassed sinus involvement, palate–sinus fistula, conjunctivitis, inflammation of the left orbital tissue, hearing loss in the left ear, and an inflammatory tumor of the left lung. Additional sinus biopsy confirmed typical granulomas with PR3-ANCA-positive serology. The destruction of the left side of the nasal cavity was present at the initial presentation, along with necrotizing tissues covering the margins of the healthy tissues (Figure 6). The medial wall of the orbit was partially preserved. Imaging confirmed the involvement of the nasal and orbital tissues with significant destruction of bony structures as well as a large tumor (90 × 47 × 42 mm) with multiple smaller ones in the right lung. The lesions in the middle face were extremely painful for the patient.

The treatment with CYC was initiated, with i.v. administration once a month. After the fifth dose, we still observed progression, so a decision to introduce an additional agent was made.

**Figure 7:**
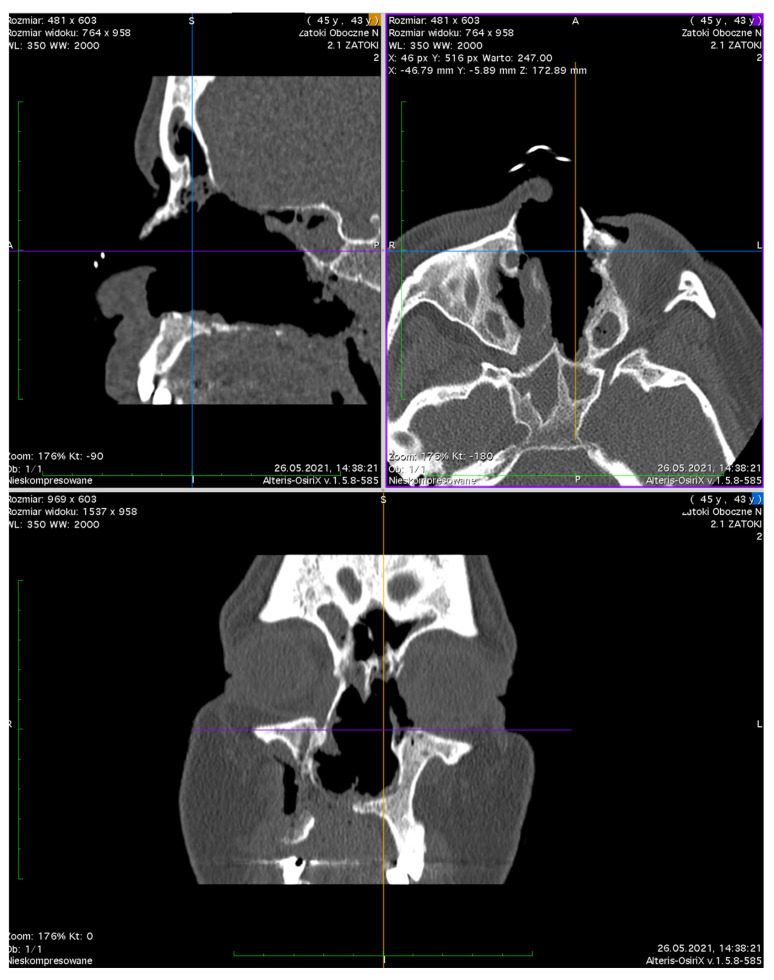
A CT scan of the head. Loss of bony and soft tissue in the middle face and left orbital walls.

**Figure 8:**
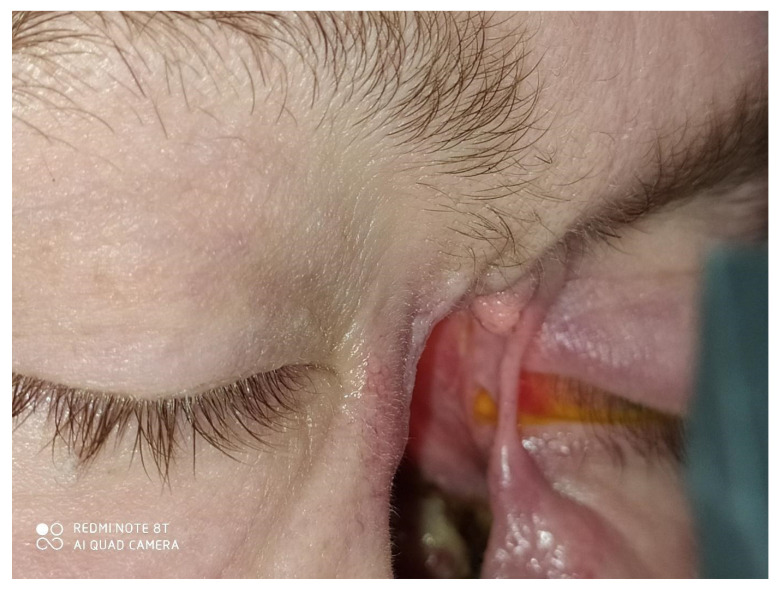
Loss of skin tissue in the median palpebral angle, loss of conjunctiva. Necrotizing tissue covered with whitish discharge. Lagophthalmos of the left eye.

**Figure 9:**
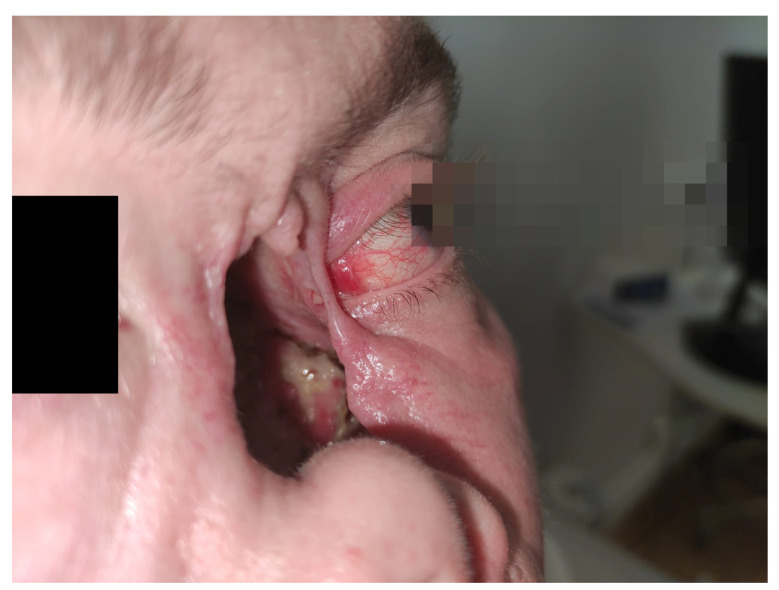
Structures of the orbit could be assessed through the collapsed nasal cavity. The following was noted in the patient: necrotic tissue at the medial wall and floor of the orbit, visible medial loss of conjunctival tissue on the orbital wall, and bare medial rectus muscle.

Computed Tomography (CT) of the orbit (Figure 7) revealed the destruction of the medial orbital wall with an inflammatory involvement of the medial rectus and inferior rectus muscles with the limitation of fat tissue in the orbit. The sinuses were open, communicating with the nasal cavity. From the clinical point of view, the structures of the orbit were seen through the nose, the muscles were bare, and the medial part of the orbit was not covered with any tissue; no conjunctiva was present (Figure 8 and Figure 9).

Moreover, the middle and lower parts of the respiratory tract were involved (lesions confirmed by bronchoscopy), and the patient presented dyspnea and chronic cough. The progression was confirmed clinically and with multiple imaging modalities, despite the intensive immunosuppressive treatment.

After six months of CYC, RTX, a biologic agent, was added to the standard scheme of therapy inducing remission, administered in a weekly dose during a 1-day inpatient clinic stay (four doses).

Following this therapy, she was reassessed by an ophthalmologist. She had diplopia when looking up, tearing, redness, and sometimes purulent discharge. Her visual acuity was logMar 0.0 in the RE and logMar 0.2 in the LE, which was involved. The left eyeball was situated slightly lower than the RE, and enophthalmos was diagnosed due to massive destruction of the medial wall and the orbital floor. No conjunctiva was present near the foci of necrosis. The sensation was impaired in the area of the lower eyelid. Bare medial and inferior rectus muscles were seen through the nasal cavity (Figure 8). The eyeball itself was well preserved but required intensive use of artificial tears.

**Figure 10:**
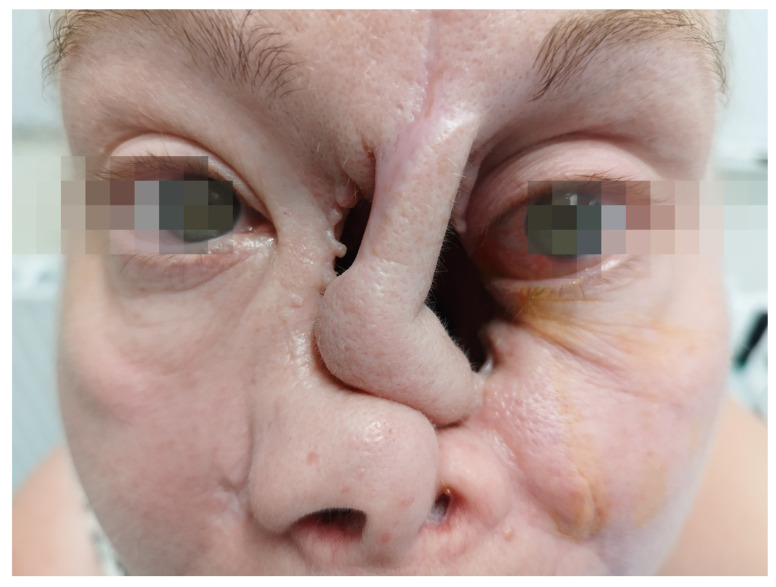
Patient after the initial step of the planned reconstructive surgery. Skin transplant partially covers the nasal cavity. The left eyeball is significantly lower with the enophthalmos of 4 mm. There is a relatively high risk of displacement of the affected eye into the orbit.

CT re-examination of facial structures revealed a severe progression in bony destruction. MTX was administered. Until now, the patient has been constantly monitored. Achieving remission is very difficult in such a severe case. As soon as remission is established, surgical reconstruction of the middle face will be performed. The outcome of the initial surgery is presented in Figure 10. It should be noted that the eyeball lost its bony support, and hypotropia and enophthalmos of 4 mm are present. Also, the lower eyelid does not have enough support, contributing to lagophthalmos and its negative consequences for the surface of the eye. From the ophthalmic point of view, the progression of the malposition of the eye and the eyelid was observed in the follow up. There is a relatively high risk of displacement of the affected eye into the left orbit.

### 3.3. Case 3

Clinical presentation includes sixth cranial nerve (CN) palsy, diplopia, central venous thrombosis with optic disc edema, and a retrobulbar mass causing compressive optic neuropathy.

We present a case of a 16-year-old previously healthy female.

In March 2019, the first symptoms of GPA included gingivitis, chronic otitis media with purulent discharge, sinusitis, peripheral paralysis of the seventh cranial nerve, salivary gland inflammation, headache, and diplopia due to the sixth CN palsy. At the initial presentation, magnetic resonance imaging of the head (MRI) was remarkable for massive inflammatory involvement of the following head structures: the pyramids of the temporal bones, airless mastoid processes, exudative sphenoid sinusitis, and bilateral ethmoid sinusitis. Based on the histopathological examination of the gingival mucosa (chronic granulomatous inflammation with giant cell reaction) and a high level of serum cANCA antibodies, a localized form of granulomatosis with polyangiitis was diagnosed in the Pediatrics Department outside our facility. The initial treatment included oral GCs (prednisolone 20 mg a day) and MMF 2 g a day, achieving partial clinical improvement.

Visual acuity was logMAR 0.0 in both eyes at that time.

Despite the therapy, new symptoms appeared after 3 months as follows: blurred vision, headaches, and increased discharge from the right auditory canal. Five intravenous doses of methylprednisolone at 1 g daily were administered. Repeated MRI showed bilateral massive involvement of the pyramidal structures of the temporal bones with complete airless mastoiditis and pansinusitis. Therefore, a relapse was confirmed, and pulses of intravenous methylprednisolone were used again (a total dose of 3 g). Treatment with MMF was considered ineffective. For this reason, a total dose of 6.5 g of CYC was introduced in a 7-month regimen with monthly administration.

Six months after the end of CYC therapy, due to a lack of improvement in sinusitis, mastoiditis, gingivitis, and deep palate ulceration, the treatment was intensified. The patient was qualified for urgent intravenous methylprednisolone (total dose: 1 g), MMF 2 g/day, and AZA 100 mg/day with a continuation of 40 mg of daily oral prednisone.

**Figure 11:**
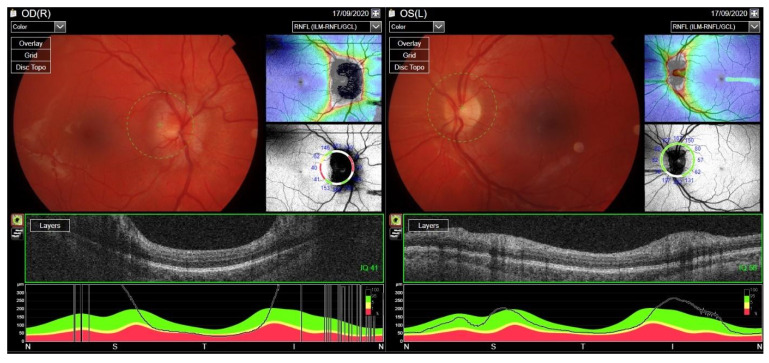
OCT RNFL image. Bilateral optic disc edema due to central venous thrombosis, more pronounced in the RE.

In September 2020, the patient developed bilateral optic disc edema RE > LE (Figure 11). The MRI of the head revealed extensive thrombosis of the CNS venous sinuses. Low-molecular-weight heparin was used, which enabled the regression of the thrombotic lesions. The patient had full visual acuity in both eyes.

**Figure 12:**
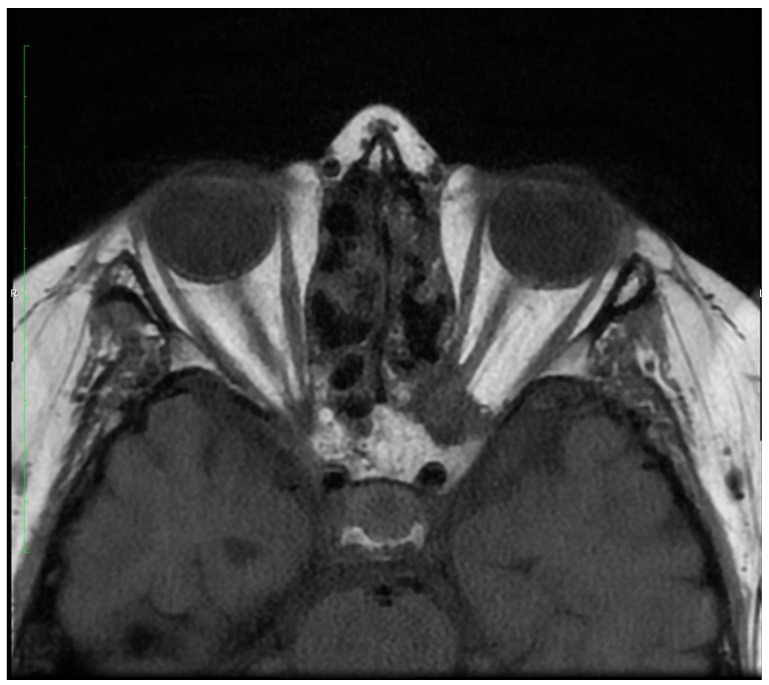
MRI of axial plane—a compressive mass in the left retrobulbar space causing the compression of the left optic nerve.

**Figure 13:**
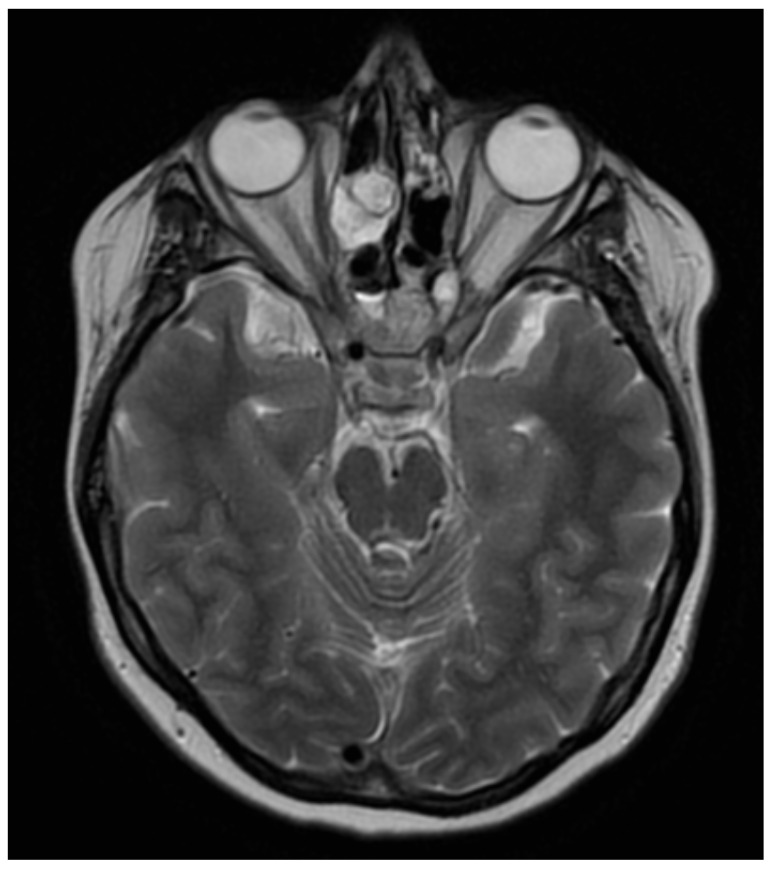
MRI of coronal plane with a massive lesion in the right ethmoid sinus cavity.

In November 2020, the patient presented with a sudden loss of vision in the LE limited to light perception due to the compression of the left optic nerve with inflammatory/granulomatous masses extending from the ethmoid (Figure 12). Methylprednisolone and immunoglobulins were administered intravenously. Due to the high activity of the disease and the high risk of vision loss in the RE, rituximab was introduced. Four weekly RTX infusions were given with a subjective improvement of vision. In the last week of the treatment, a fistula formed in the ethmoid lamina, causing the rhinorrhea of the cerebrospinal fluid through the right nasal passage (Figure 13). Subsequently, RTX treatment was complicated by the reactivation of opportunistic infections: CMV and HSV1 (high transaminases). Therefore, ganciclovir was introduced.

A lack of remission and a high risk of vision loss in the RE caused CYC reintroduction according to the EUVAS protocol, prednisone was increased to 60 mg a day, and immunoglobulins were administered intravenously (0.4 mg/kg b.w.). In March 2021, the infusions of CYC were discontinued due to the development of COVID-19 pneumonia. Multiple fractures of the thoracic and lumbar spine were diagnosed, and stabilization was applied using the Jewett orthopedic corset.

After the resolution of the infection, CYC was administered again.

**Figure 14:**
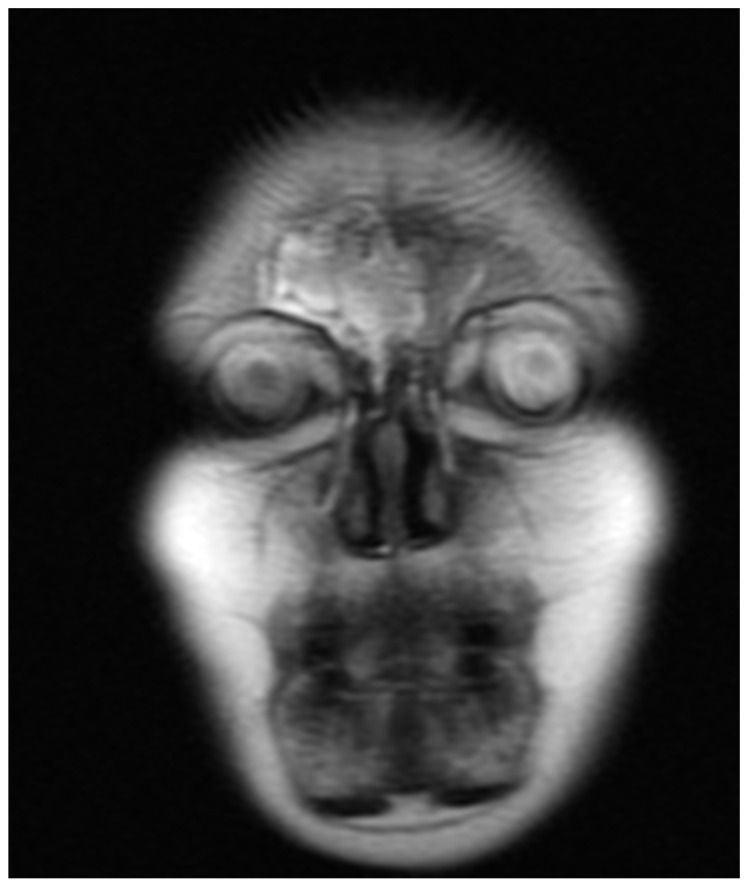
MRI of the head and axial plane revealed frontal sinus involvement, more severe on the right side.

**Figure 15:**
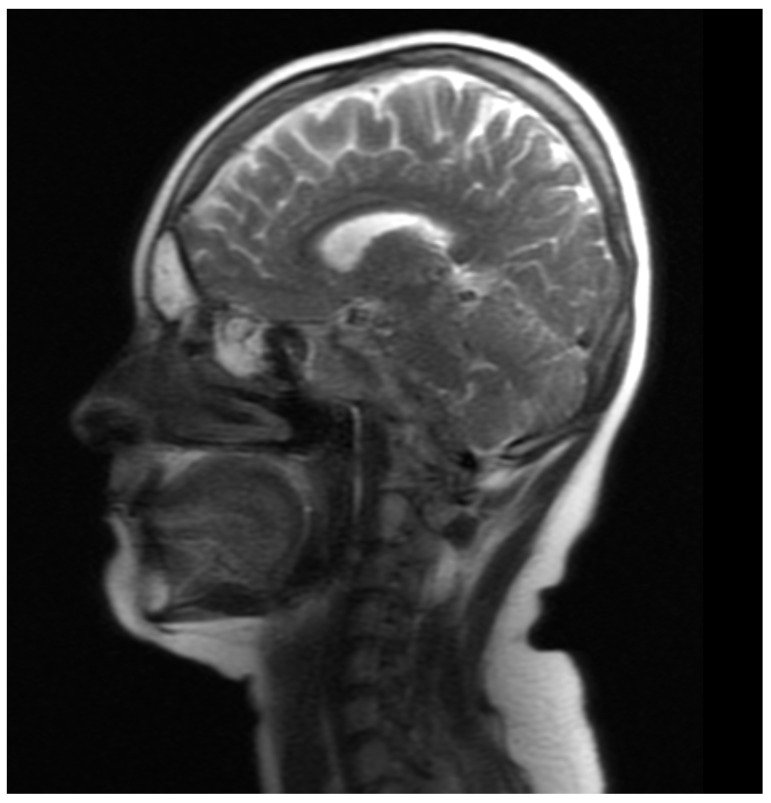
MRI of the head (sagittal plane) revealed frontal and ethmoid sinus involvement.

In March 2022, during another hospitalization, laboratory tests revealed the following: an erythrocyte sedimentation rate of 15 mm after 1 h, CRP 2.3 mg/dL, normal liver and kidney function parameters, slight neutrophilic leukocytosis (secondary to steroid therapy), IgA, IgG, and IgM within normal limits. Chest X-ray was positive for single-banded changes in the inferior lobe of the right lung. Apart from those findings, the X-ray results were unremarkable. However, head MR showed smooth thickening of the brain dura mater, revealing new inflammatory lesions in the frontal sinuses (Figure 14 and Figure 15).

**Figure 16:**
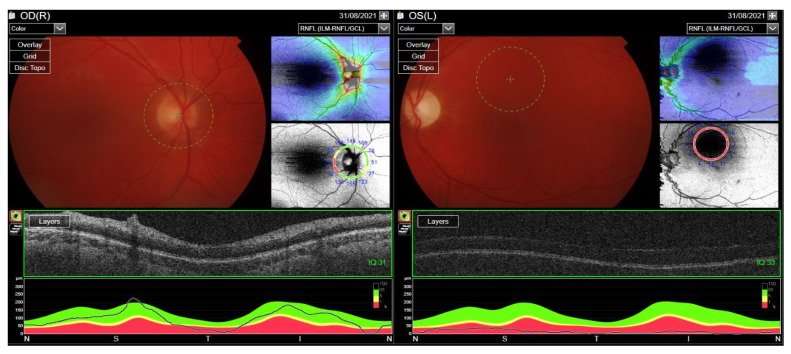
Fundus OCT and RNFL image. Optic disc pallor following compressive neuropathy in the LE. Difficulty with fixation due to light perception in the LE.

Treatment with RTX was reintroduced. Cyclosporine dose was increased to 200 mg/day and methotrexate up to 30 mg/week. After the RTX therapy course, the ocular assessment revealed a quiet eye with left compressive optic neuropathy (a pale disc with RNFL atrophy) and bilateral posterior subcapsular cataracts (Figure 16). The final visual acuity was logMar 0.0 in the RE and light perception in the LE.

### 3.4. Case 4

Clinical presentation includes orbital involvement, retrobulbar mass, exophthalmos, diplopia, and eyelid abscess.

A 32-year-old man had already been diagnosed with granulomatosis with polyangiitis for 8 years.

**Figure 17:**
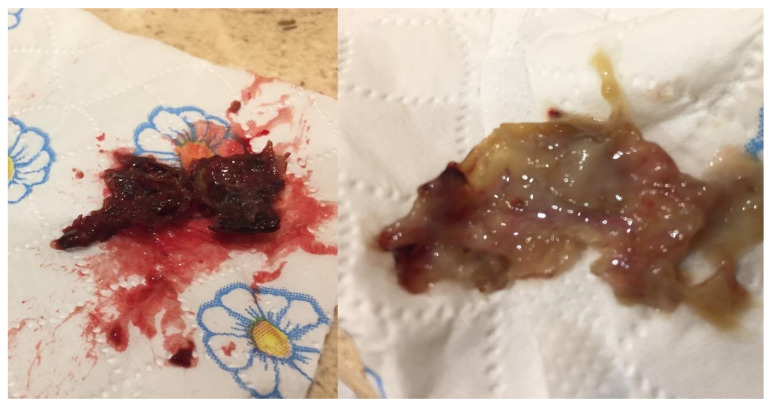
Typical nasal discharge in a GPA patient.

The first symptoms of GPA included epistaxis, chronic atrophic rhinitis, nasal ulcers, granulomatous necrotic nasal septum deviation (confirmed by histopathology), skin ulcers, and three pulmonary infiltrates with a diameter of about 2.5 cm detected in a pulmonary X-ray (Figure 17). No ocular involvement was diagnosed. Under the circumstances, an autoimmune disorder was suspected, and the patient was referred to the Rheumatology Department. After an extensive work up, considering the otorhinolaryngology manifestations, pulmonary lesions, skin ulcers, high blood levels of inflammatory markers, and positive serum c-ANCA, the diagnostic criteria for active systemic GPA were met.

The induction therapy of choice was an intermittent pulse therapy (a 12-month regimen, monthly administration) with intravenous methylprednisolone (total dose: 6 g [a single dose was 500 mg i.v.]) and intravenous CYC (total dose: 9.6 g [a single dose was 800 mg i.v.]) plus oral prednisolone (starting dose 40 mg/day, with slow tapering every month). After achieving remission, the treatment was switched to maintenance therapy (oral prednisolone with constant tapering rate and AZA 100 mg/day).

**Figure 18:**
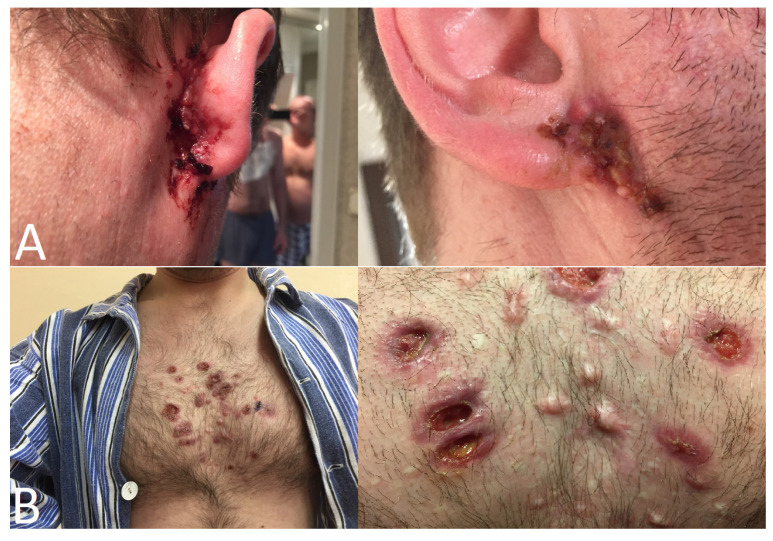
(**A**) Skin lesions on the on the face (behind and in front of the right ear) and (**B**) on the chest.

After two years of maintenance therapy, a relapse was diagnosed with the occurrence of a new infiltration in the right lung and ulceration of the facial skin (Figure 18A). Again, the conventional initial treatment included high-dose GCs and CYC for at least 9 months until remission, followed by maintenance therapy with AZA and oral prednisolone.

**Figure 19:**
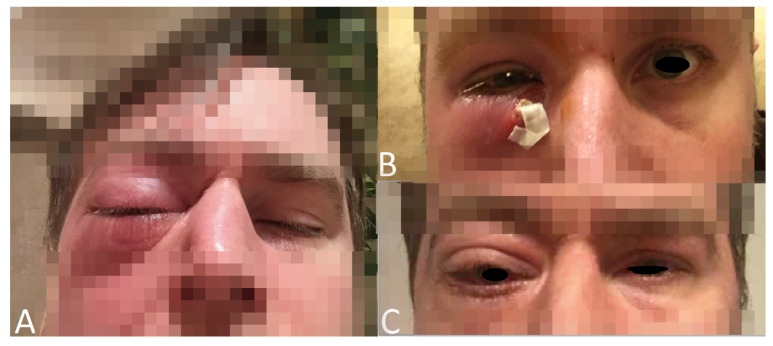
(**A**) Right eyelid edema due to an abscess in the lower eyelid. (**B**) The right lower eyelid after surgical evacuation of the abscess. (**C**) Improvement of the edema.

Seven years after GPA diagnosis (prednisolone 15 mg/day, azathioprine 100 mg/day), the first ocular symptom appeared: a massive abscess of the lower eyelid with swelling and hyperemia of both eyelids of the RE (Figure 19). It resolved following abscess drainage and systemic antibiotic therapy.

A year later, despite maintenance treatment, the patient was diagnosed with an exacerbation of the disease in the form of ulcerative skin lesions on the chest and face (Figure 18) and the obstruction of the lacrimal ducts on the right side with redness and tearing. General treatment was modified by a rheumatologist by adding subcutaneous MTX (30 mg). After clinical improvement, a successful external dacryocystorhinostomy (DCR) was performed on the right lacrimal ducts. In the postoperative course, standard systemic treatment with clindamycin 300 mg three times a day was applied, and topical tobramycin and Betadrin/naphazoline drops were instilled three times a day.

**Figure 20:**
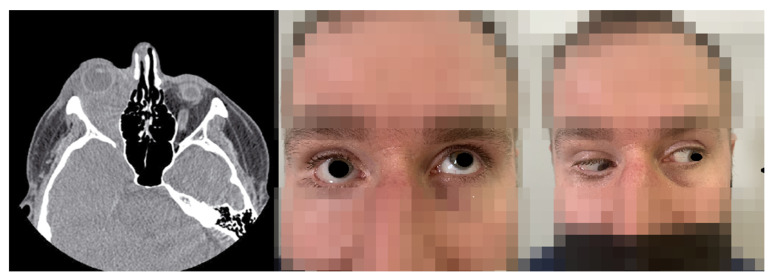
CT of the head and orbit. The image revealed a retrobulbar massive lesion on the right side. Proptosis of the right eye and RE motility restriction.

Five months later, in March 2018, the 32-year-old patient at that time, presented in the Ophthalmology Department with the following complaints in the RE: mild retrobulbar discomfort, proptosis, mild eyeball mobility restriction, and epiphora (Figure 20). On examination, the best corrected visual acuity was logMAR 0.2 in the RE and logMAR 0.0 in the LE. Intraocular pressure values were within normal limits bilaterally. The anterior segment proved to be normal in the LE. However, proptosis, upper and lower lid swelling, ptosis, slightly decreased ocular motility, mild conjunctival hyperemia, and chemosis were noted in the RE. CT scans and ultrasonography of the orbits showed an extensive intraorbital infiltration, spreading from the myofascial extraocular muscle cone towards the eyeball, contributing to its protrusion (Figure 20). Histopathological evaluation revealed a proliferation of small and medium-sized vessels with an intense, mixed inflammatory infiltrate.

Because of the rapidly progressing exophthalmos of the RE accompanied by severe pain and a decrease in visual acuity to logMAR 0.8, the patient was qualified for urgent surgical decompression through lateral canthotomy of the right orbit with the excision of the intraorbital pseudotumor in the Maxillofacial Department. Following surgical treatment, visual acuity improved to logMAR 0.3.

A month later, during an examination in the Ophthalmology Department, a CT scan revealed the modeling of the intraorbital structures on the right side by inflammatory granulation tissue, resulting in proptosis. Additionally, limited eyeball mobility in all directions, swelling and redness of the eyelids, conjunctival chemosis, secondary ocular hypertension (20.0 mmHg), and compressive optic neuropathy were observed.

**Figure 21:**
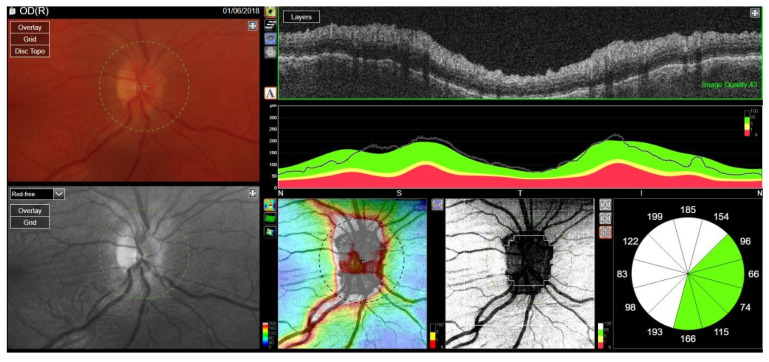
OCT RNFL. Optic disc edema in the right eye caused by retrobulbar granulomatosis.

BCVA in the RE decreased to logMAR 0.5, and disc edema was found on an ophthalmoscopic examination, confirmed in spectral-domain optical coherence tomography (OCT) of the optic nerve scans, illustrating an increase in the retinal nerve fiber layer (RNFL) thickness in the superior quadrant of the RE and scotoma in the lower sector of standard automated perimetry (Figure 21). The patient was qualified for urgent intravenous methylprednisolone (total dose: 2.625 g) and intravenous two-week administration of CYC (total dose: 1.2 g) with continuous oral prednisone therapy at a dose of 80 mg daily. Due to the lack of clinical response to CYC, treatment with RTX was initiated at a dose of 375 mg/m^2^/week for 4 weeks. The patient presented a good clinical response, with a complete resolution of the lesion confirmed radiologically 4 months after starting RTX.

Compressive massive lesion.

## 4. Discussion

The presented case report series illustrates the importance of considering GPA as the underlying diagnosis of a variety of ocular symptoms. Our publication is distinguished by numerous photographic records of ocular and extraocular signs. The characteristics of the patients are summarized in Table 1. The age of our patients ranged from 16 to 43 years. The analysis included two women and two men. The serological work up revealed positive c-ANCA antibodies. One of the patients presented with numerous multiorgan symptoms, but it was an ophthalmologist who diagnosed GPA based on the ocular symptoms (case no. 1). Symptoms associated with orbital involvement occurred in three patients in our series. All patients had unilateral manifestations. All patients received CYC as the first line of treatment, but only one patient achieved complete remission of ocular symptoms. In the remaining patients, ophthalmic improvement was observed after the administration of RTX (the second line of treatment). Compressive optic neuropathy is the most severe complication of the ocular form of GPA. It occurred in two cases and led to irreversible visual loss in one case (case no. 3). Urgent surgical decompression of the orbit through lateral canthotomy with the excision of the intraorbital pseudotumor prevented permanent vision loss in case no. 2.

Following the diagnosis, GPA patients are responsive to immunosuppressive therapy with or without RTX and may achieve near-complete resolution of ocular symptoms. In our series, one patient suffered from permanent visual loss due to compressive optic neuropathy.

Ocular manifestations in the course of the disease were seen in ca 26–50% of patients with GPA [15,16,17]. In 15.6–19.3% of cases, ocular symptoms constituted the first sign of the disease [15,18,19,20,21]. Thus, an ophthalmologist should consider the diagnosis of GPA, especially in cases where other features of the disease, such as pulmonary or renal disease, are absent.

The exact cause of GPA is not well understood. The etiopathogenesis has been credited to the presence of ANCA. GPA is characterized by an inflammatory reaction pattern (necrosis, granulomatous inflammation, and vasculitis). GPA may affect every structure of the eye: the orbit, sclera, episclera, cornea, conjunctiva, eyelids, nasolacrimal system, optic nerve, retina, and the uvea. The most common manifestations include conjunctivitis/episcleritis (44.5%) and scleritis (20.9%), followed by orbital pseudotumor [15]. In multivariable analysis, the sino-nasal involvement of GPA was associated with dacryocystitis, lacrimal duct obstruction, and retro-orbital disease, but lesions occurred via necrosis or granulomas directly penetrating tissues [15]. In addition, patients are at risk of ocular complications related to drug toxicity. Cataract is the most common side effect associated with ocular drugs.

Ocular symptoms of GPA may be the first manifestation of the disease. Therefore, ophthalmologists should pay attention to the differential diagnosis in patients who do not respond to standard therapy. The American College of Rheumatology established the following classification criteria for GPA: (1) urinary sediment showing red blood cell casts or more than five red blood cells per high power field, (2) abnormal findings on chest radiograph, (3) oral ulcers or nasal discharge, and (4) granulomatous inflammation on biopsy [22]. The presence of two out of four of these criteria is 88.2% and 92% sensitive and specific, respectively [22].

Routine laboratory tests include complete blood count with differential, serum albumin and total protein, electrolytes, serum creatinine, urinalysis, 24 h urine protein, erythrocyte sedimentation rate (ESR), and C-reactive protein (CRP). ANCA testing and selective biopsy of involved tissues yield the most specific findings. Although 90% of patients with active diseases are c-ANCA-positive, 40% of patients with organ-limited diseases may be negative [23]. Imaging diagnostics, such as orbital and sinus CT (with or without contrast), MRI, orbital and eye ultrasound, may be helpful in the diagnosis and may reveal sinus mucosal thickening or opacification, nasal septum perforation, bone damage, nodules, masses and cavitary lesions caused by GPA. Retinal vasculitis can be confirmed in fluorescein angiography.

The treatment of GPA involves the use of immunosuppressive agents in a variety of combinations. Ophthalmologists do not treat GPA themselves, as it is a very severe, mostly multiorgan, life-threatening disease exceeding ophthalmological training; they usually do not have suitable experience. Their role involves being a consulting physicians. Patients should be immediately referred to tertiary centers, which treat such patients on a daily basis. Nonetheless, the role of ophthalmologists is very important, as they can be the first to diagnose the condition and refer the patient to a suitable facility. Moreover, during the disease, they assess the severity of ocular manifestations and guide the leading physician in case of relapses or confirm stability.

The treatment of AAV includes two phases: the induction phase and the maintenance phase, with the commonly used agents being cyclophosphamide, glucocorticoids, rituximab, azathioprine, methotrexate, and plasmapheresis if indicated.

Additional surgical management may be useful to decompress the orbit in cases of orbital GPA with severe pain, proptosis, or compressive optic neuropathy. In the case of a risk of perforation in the course of scleritis, a scleral graft, amniotic patch, and/or tarsorrhaphy should be considered. Patients should be advised not to rub their eyes and avoid injury. For midface or DCR reconstructive surgery, it is generally accepted that a patient with GPA should be in remission for 6 months or more, and maintenance immunosuppressive therapy should include low-dose immunosuppressants. The ongoing treatment needs to be maintained in the periprocedural period [16]. Before surgery, the parameters of inflammation and the presence of cANCA, creatinine, and glucose levels in the blood serum should be verified. Low levels of cANCA antibodies or their absence increase the chance of successful surgery and are associated with a low risk of GPA relapse. The presence of diabetes mellitus may affect the healing process. Therefore, it is best to qualify patients with normal glycemia for reconstructive surgery.

## Figures and Tables

**Table 1:** Characteristics of the patients with ocular manifestations.

Characteristic	Case 1	Case 2	Case 3	Case 4
Age (years)	40	43	20	32
Age at diagnosis	40	40	16	24
Sex	Male	Female	Female	Male
Ethnicity	Caucasian	Caucasian	Caucasian	Caucasian
ANCA serology	cANCA-positive pANCA-negative	cANCA-positive pANCA-negative	cANCA-positive pANCA-negative	cANCA-positive -pANCA negative
Type of eye disease	Peripheral ulcerative keratitis Retinal vasculitis/Retinal hemorrhage/exudates Necrotizing scleritis	Orbital disease enophthalmos	Optic nerve involvement and bilateral posterior subcapsular cataracts	Retrobulbar mass and proptosis, Dacryocystitis and lacrimal duct obstruction
Timing of eye involvement	Systemic involvement first	Both ocular and systemic involvement at the onset	Systemic involvement first	Systemic involvement first
Symptoms at diagnosis	Severe skin ulceration, lung and renal involvement	Orbital, sinus, lung involvement	Mastoiditis, pansinusitis, gingivitis, deep palate ulceration	Nasal and lung involvement; severe skin ulceration
Treatment	CYC (6 months)	CYC (6 months), RTX was started due to the lack of clinical response	CYC (6 months), RTX was administered due to the lack of clinical response	CYC, RTX was administered twice due to the lack of clinical response
Outcome	Remission	Progressive	Progressive	Remission
VA in the affected eye [logMAR]	0.0	0.2	Light perception	0.0

## Data Availability

Not applicable.
